# Women's views on screening for Type 2 diabetes after gestational diabetes: a systematic review, qualitative synthesis and recommendations for increasing uptake

**DOI:** 10.1111/dme.14081

**Published:** 2019-07-22

**Authors:** R. A. Dennison, R. A. Fox, R. J. Ward, S. J. Griffin, J. A. Usher‐Smith

**Affiliations:** ^1^ Primary Care Unit Department of Public Health and Primary Care University of Cambridge Cambridge UK; ^2^ School of Clinical Medicine University of Cambridge Cambridge UK; ^3^ MRC Epidemiology Unit University of Cambridge Cambridge UK

## Abstract

**Aim:**

Many women do not attend recommended glucose testing following a pregnancy affected by gestational diabetes (GDM). We aimed to synthesize the literature regarding the views and experiences of women with a history of GDM on postpartum glucose testing, focusing on barriers and facilitators to attendance.

**Methods:**

We systematically identified qualitative studies that examine women's experiences following GDM relating to glucose testing (diabetes screening) or experience of interventions to promote uptake of testing. We conducted a thematic synthesis to develop descriptive and then analytical themes, then developed recommendations to increase uptake based on the findings. We evaluated the quality of each study and the confidence that we had in the recommendations using published checklists.

**Results:**

We included 16 articles after screening 23 160 citations and 129 full texts. We identified four themes of influences relating to the healthcare system and personal factors that affected both ability and motivation to attend: relationship with health care, logistics of appointments and tests, family‐related practicalities and concern about diabetes. We developed 10 recommendations addressing diabetes risk information and education, and changes to healthcare systems to promote increased attendance at screening in this population, most with high or moderate confidence.

**Conclusions:**

We have identified a need to improve women's understanding about Type 2 diabetes and GDM, and to adjust healthcare provision during and after pregnancy to decrease barriers and increase motivation for testing. Encouraging higher uptake by incorporating these recommendations into practice will enable earlier management of diabetes and improve long‐term outcomes.

## What's new?

- There is a need to increase the number of women attending glucose testing after gestational diabetes. Higher attendance will enable earlier diagnosis and management of diabetes and improve long‐term outcomes.
- This is the first qualitative review focusing on barriers and facilitators to screening attendance.
- We found that factors could affect either mothers’ motivation or opportunity to attend.
- Some influences related to the healthcare system (relationship with health care and logistics of the appointment and test), whereas others were personal (concern about diabetes and family‐related practicalities).
- We developed 10 recommendations to increase screening attendance based on the barriers and facilitators identified.

**What's new?**

- There is a need to increase the number of women attending glucose testing after gestational diabetes. Higher attendance will enable earlier diagnosis and management of diabetes and improve long‐term outcomes.
- This is the first qualitative review focusing on barriers and facilitators to screening attendance.
- We found that factors could affect either mothers’ motivation or opportunity to attend.
- Some influences related to the healthcare system (relationship with health care and logistics of the appointment and test), whereas others were personal (concern about diabetes and family‐related practicalities).
- We developed 10 recommendations to increase screening attendance based on the barriers and facilitators identified.

## Introduction

Gestational diabetes (GDM) is an increasingly common disorder, with ~ 14% of pregnancies affected worldwide 1. In addition to increasing the risks of pregnancy complications that affect both mother and baby, it is associated with increased risk of cardiometabolic disease after pregnancy; this is often overlooked 2. Specifically, women with GDM are eight times more likely to develop Type 2 diabetes than unaffected women 3, and this risk is highest during the first 5 years postpartum 4. Along with diabetes risk factors such as high body mass index and older age, maternal and pregnancy‐related factors such as poorer pregnancy glycaemic control that needs to be managed with insulin have been suggested to further increase the risk of developing diabetes after GDM 5, 6.

National and international guidelines recommend that pregnant women are screened for glucose abnormalities at 1–3 months postpartum to exclude persisting diabetes 7, 8. Women should then be screened regularly according to previous test results in order to monitor glucose levels and identify those at highest risk of progressing to diabetes 7, 8. Earlier detection of Type 2 diabetes and effective management of ‘pre‐diabetes’ decreases exposure to hyperglycaemia and hence reduces risk of longer‐term complications and all‐cause mortality 9. There is currently variation between guidelines about which screening tests and schedules to use. For example, the American Diabetes Association (ADA) recommends using the 75‐g oral glucose tolerance test (OGTT) at the first postpartum test, followed by either a fasting plasma glucose (FPG) test, OGTT or HbA_1c_ at least every 3 years 7. In 2015, the National Institute for Health and Care Excellence (NICE) advised that women in the UK should be screened using FPG postpartum followed by annual HbA_1c_ testing, and should not be routinely offered an OGTT 8.

Frequency of postpartum screening varies by population but remains suboptimal; many studies report just 50% uptake 10, 11, 12, 13. Younger women with other children and of lower socio‐economic status attend less frequently, particularly if they received little perinatal care or their GDM was managed by diet alone 13. Not all women who access postpartum care after GDM receive appropriate diabetes screening 13. These observations are consistent with lower long‐term engagement in behaviour change interventions in women with GDM compared with other populations 14, highlighting the difficulty engaging this population in interventions aimed at reducing diabetes risk. A systematic review of both qualitative studies and surveys found that healthcare seeking after GDM can be constrained by the maternal role (meaning prioritizing the needs of children and constraints associated with childcare), failures of the healthcare system and women's perspectives towards testing 15. However, only studies published up to 2013 were included and general care, rather than glucose testing, was considered.

In light of recently published studies about screening plus changing guidelines for gestational and Type 2 diabetes diagnosis and management 7, 8, 16, 17, 18, 19, we have systematically synthesized the literature up to September 2017 regarding the views and experiences of women with a history of GDM on follow‐up glucose testing. We focused in particular on barriers and facilitators to attendance. Furthermore, we have developed recommendations to adjust testing protocols or inform interventions for improving long‐term follow‐up based on the findings.

## Methods

Details of the protocol for this systematic review were registered on PROSPERO (CRD42018092386; http://www.crd.york.ac.uk/prospero).

Methods for the systematic search and analysis were the same as those used for a parallel review, synthesizing views on a healthy lifestyle after a pregnancy affected by GDM 20.

### Search strategy

In brief, the search strategy shown in Table S1 was used to search CINAHL, the Cochrane Library, Embase, MEDLINE and PsychINFO electronic databases. This was developed for a group of literature reviews concerning GDM. There were no language or other restrictions. We also screened the reference lists of included studies for citations not identified by this search.

### Study selection

We included peer‐reviewed journal articles that examine women's experiences following GDM relating to postpartum glucose tolerance testing or Type 2 diabetes screening, or experience of interventions to promote screening. All qualitative and mixed methods studies were eligible. We excluded studies exclusively reporting views of healthcare providers and about postpartum lifestyle in order to focus on screening.

After removing duplicates, R.D. or R.W. assessed all titles and abstracts against these selection criteria. We used an overlap of ~ 10% to ensure agreement between decisions. Any differences were discussed with all authors and the selection criteria were refined and elaborated accordingly. R.D. and R.F. then acquired full text articles and reassessed them against these criteria, again with 10% overlap.

### Quality assessment

R.F. used the Critical Appraisal Skills Programmes (CASP) checklist for qualitative research 21 to assess the quality of the qualitative research in each study, with discussion with R.D. Scores of 0, 0.5 and 1 were awarded for answering ‘no’, ‘unclear’ and ‘yes’ to each of the 10 questions. We did not exclude studies based on quality in order to make use of all available information. We did, however, take the quality of the studies into account when developing our themes and recommendations, and assessed the contribution of lower quality studies to the findings in Tables S3 and S4.

### Qualitative synthesis

**Figure 1:**
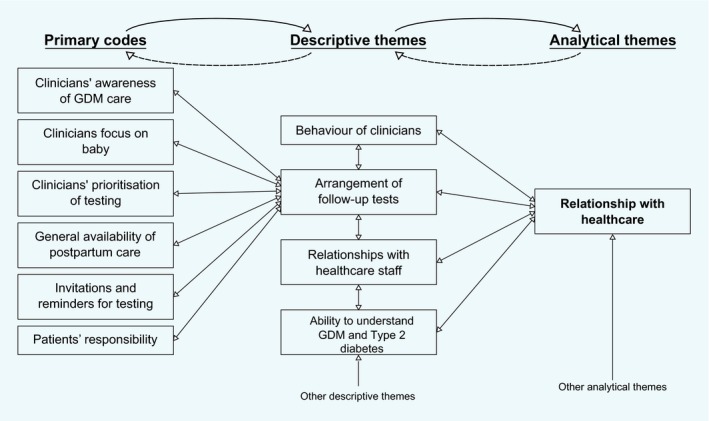
Example of the development of the analytical theme ‘Relationship with health care’ within the thematic synthesis. Not all codes were presented for simplicity. GDM, gestational diabetes.

We conducted a thematic synthesis 22 with the aid of NVivo 11. Text and tables labelled as ‘Results’ (or equivalent) that resulted from qualitative methods were used as data. After familiarizing ourselves with the data, R.D. and R.F. formed a coding frame and used this to develop descriptive themes. Both authors extracted and coded data, including independently coding a subset of papers at multiple stages to check consistency. In the second stage, concepts were translated from one study and category to another by making summaries and comparisons, and new concepts developed as illustrated in Fig. 1. R.D. and R.F. considered these independently, then together and finally refined the analytical themes through discussion with the wider research team.

We have presented illustrative quotations from the original studies as part of our explanation of the analytical themes to allow the primary data to be considered. We specify whether the quotations were from screened or unscreened women if this was included in the primary data. We have considered our perspectives on the analysis and results as clinical or non‐clinical researchers based in the UK. R.D. has undertaken postgraduate training in public health and completed the research as part of her doctoral studies; R.F. is a medical student; R.W. is an academic general practice registrar; and S.G. and J.U‐S. are both academic general practitioners (GPs) with qualitative research experience.

### Recommendations for promoting screening

From the analytical findings, we developed recommendations that aimed to address the behaviours or beliefs that hindered screening attendance and to make use of facilitators. We aligned each recommendation with the standardized behaviour change technique (BCT) taxonomy (v1) to enable greater consideration of the mechanism by which the recommendations could have an effect 23. We used the Grading of Recommendations Assessment, Development and Evaluation‐Confidence in Evidence from Reviews of Qualitative research (GRADE‐CERQual) approach to evaluate our confidence in each of these recommendations 24. GRADE‐CERQual considers the relevance, coherence, adequacy and methodological limitations of data contributing to each recommendation, therefore informing our confidence in its effectiveness.

## Results

**Figure 2:**
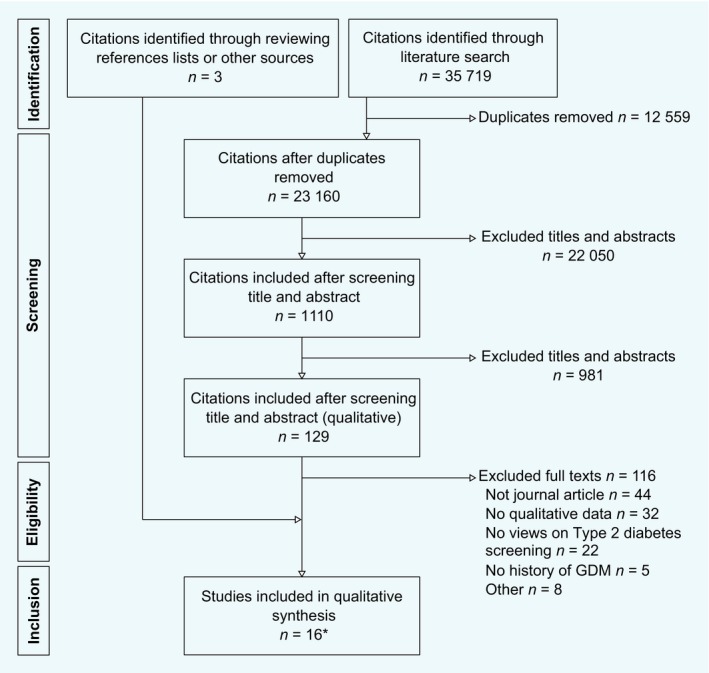
PRISMA diagram showing number of studies included at each stage of the literature review. *Two of these publications report the same set of interviews using different approaches to the analysis.

We included 16 qualitative papers after screening 23 160 citations and reviewing 129 full texts (Fig. 2). Table 1 shows the characteristics of these studies. Two papers published by Rafii *et al*. in 2017 reported data from the same set of interviews but used different analysis methods 25, 26. The median number of participants was 22 (IQR 12–31) and 746 postpartum women are represented overall. Some 53% of these participants attended testing (97 of 184, based on seven studies reporting attendance). All but one used interviews, which were most frequently conducted face‐to‐face. Most were set in high‐income countries and some recruited minority populations; where mixed populations were recruited, often over half of participants were White European. Average age was ~ 35 years (range 24–56 years). Where reported, the majority of each population was married; use of insulin during pregnancy, family history of diabetes and being overweight were common. Views towards the first postpartum test or general testing were considered and, correspondingly, data were collected between 6 weeks and 9 years after pregnancy.

**Table 1:** Characteristics of the studies included in the qualitative synthesis

Study (first author and year)	Sample size (number screened)	Setting (country)	Screening considered	Study aim(s) relevant to this analysis	Recruitment method	Participant inclusion criteria	Method of data collection	Time of data collection*	CASP rating (out of 10)
Soares 2006 27	56 (unclear)	Brazil	First postpartum programme visit (up to 60 days)	Discuss prevention of Type 2 diabetes after GDM	Women who were part of a hospital‐based diabetes care programme	hGDM 1997–2003, controlled fasting glycaemia > 95 mg/dl during gestation or > 2 Type 2 diabetes risk factors, live in Metropolitan Region of Belo Horizonte	Interviews	3–9 years postpartum	3.5
Bennett 2011 35	22 (6)	USA	First postpartum OGTT	Explore experiences, perspectives, and perceived barriers to and facilitators of postpartum follow‐up care after GDM	Consecutive sampling of women in third trimester from high‐risk obstetric clinic	hGDM, English‐speaking, insurance coverage during and beyond postpartum visit	Face‐to‐face and telephone interviews	6–8 weeks postpartum	8.5
Sterne 2011 28	88 (47)	Australia	First postpartum OGTT	Examine barriers, facilitators and potential facilitators to attendance at postpartum diabetes screening after recent GDM	Identified from a hospital database	GDM outpatient care at Logan Hospital, Meadowbrook, Queensland 2006–2007, ≥ 18 years old, no history of Type 1 or Type 2 diabetes	Telephone interviews	~ 1.5–3 years postpartum	5.5
Lie 2013 29	35 (NR)	UK	First postpartum OGTT and annual testing	Explore views on postnatal lifestyle change to prevent Type 2 diabetes to inform development of intervention approaches	Purposive then theoretical sampling (contacted by diabetes obstetric clinic staff while attending appointments or from hospital records)	hGDM within 2 years, English‐speaking, ≥ 16 years old, successful pregnancy outcome, received antenatal care at specified sites, able to consent	Face‐to‐face interviews	Within 2 years postpartum	8.0
Abraham 2014 30	10 (3)	USA	General screening after GDM	Explore lived experiences of women in rural communities with GDM and gain insight into low screening rates	Purposive sampling and a snowball approach via obstetric and healthcare provider offices	hGDM within 5 years, ≥ 18 years, reside in a county eligible for rural community grants, not since developed Type 2 diabetes	Interviews (face‐to‐face and telephone)	Between 2 and 5 years	7.0
Morrison 2014 39	393 (NR)	Australia	General screening after GDM	Describe reflections on the experience of GDM‐pregnancy	Identified from NDSS database and contacted by mail	hGDM within 3 years, ≥ 18 years old at time of registration, not residing in a Queensland postcode†	Questionnaire with free text open‐ended questions	Within 3 years postpartum (mean 1.8 ± 0.7)	6.5
Paez 2014 38	22 (17)	USA	First postpartum OGTT/FPG and annual testing	Explore what helps and hinders diabetes testing after GDM	Women not tested and those that were tested as part of ADAPT, recruited from a multispecialty group medical practice after a GDM pregnancy from medical records	GDM in most recent pregnancy, ≥18 years old, patients of HVMA, no history of Type 1 or Type 2 diabetes, internet/telephone access, no significant mental health disorders, physician approved participation	Survey and telephone interviews	6 months to 4.5 years postpartum	8.0
Kilgour 2015 33	13 (7)	Australia	First postpartum OGTT	To explore and assess women's communication experiences of postnatal GDM follow‐up, and interpret them with CAT	Theoretical sampling from clinics and wards at a major maternity tertiary referral hospital	hGDM, shared maternity care	Face‐to‐face interviews‡	12–16 weeks postpartum	9.0
Nielsen 2015 31	7 (7)	Denmark	General screening after GDM	Understand experience of GDM care and how this influenced participation in follow‐up screening	Random selection of women with previous GDM eligible at Aalborg University Hospital	hGDM 2010–2012, first GDM pregnancy, representative of the hospital registered population	Face‐to‐face interviews	1–2 years postpartum	10.0
Bernstein 2016 36	27 (NR)	USA	General screening after GDM	Barriers and facilitators to testing and referral to testing (four domains: intervention attributes, individual characteristics, inner context and outer context)	Convenience sample of women in an urban safety net hospital in third trimester	In third trimester of a GDM pregnancy	Face‐to‐face interviews	10–14 weeks postpartum	6.5
Campbell 2017 37	7 (NR)	Australia	General screening after GDM	Enablers and barriers influencing screening after GDM in Australian Indigenous women and how screening might be improved	Recruited by health service staff and project flyers in waiting area of health service	hGDM, Indigenous	Face‐to‐face interviews	<5 years for 4 women, >5 years for 3 women	9.0
Pennington 2017 32	16 (NR)	Australia	General screening after GDM	Investigate factors influencing engagement with diabetes preventative care (barriers and enablers)	Purposive sampling (approached or advertisements at general practices and MCHN centres)	hGDM	Face‐to‐face and telephone interviews	NR	8.0
Rafii 2017 25, 26	22 (unclear§)	Iran	First postpartum OGTT/FPG	Explore Iranian women's experiences of on obstacles of postpartum diabetes screening	Purposeful then theoretical sampling from (governmental and private) hospital records after GDM	GDM diagnosis by hospital records, delivered > 6 months before interview	Face‐to‐face interviews	Mean 11.9 ± 4.8 months postpartum	7.5 and 9.5, respectively
Svensson 2017 40	5 (NR)	Denmark	General screening after GDM	Examine the experience of transition from a GDM‐affected pregnancy to postpartum	Random sampling (sent invitation letters via the hospital patient registry and telephoned)	hGDM, recently delivered at the hospital	Face‐to‐face interviews	Between 3 and 5 months postpartum	7.5
Zulfiqar 2017 34	23 (unclear)¶	Australia	First postpartum OGTT and annual testing	Explore barriers and facilitators to following long‐term healthy lifestyle recommendations, and whether there were differences between overseas‐born‐ and Australian‐born‐women	Women managed by a hospital DIP Service who attended a GDM‐related health education programme	hGDM, English‐speaking, live singleton delivery, not pregnant or since developed Type 2 diabetes	Face‐to‐face interviews	More than 3 years postpartum	7.5

* In reference to/since GDM pregnancy; studies collected data once postpartum unless otherwise specified.

† Due to a concurrent study.

‡ Face‐to‐face interview is implied.

§ Rafii 25 reported 10 of 22, whereas Rafii 26 reported 11 of 22 attended screening.

¶ ‘Almost all’ had 6 weeks, ‘most’ had first year, ‘few’ had second year tests.

ADAPT, Avoiding Diabetes After Pregnancy Trial; CASP, Critical Appraisal Skills Programme checklist; DIP, Diabetes in Pregnancy; FPG, fasting plasma glucose; (h)GDM: (history of) gestational diabetes; HVMA, Harvard Vanguard Medical Associates; MCHN, maternal and child health nurse centres; NDSS, National Diabetes Service Scheme; OGTT, oral glucose tolerance test.

We found most of the studies to be good quality (mean CASP score 7.6/10), as detailed in Table S2. Two studies scored below 6/10 because they did not report use of rigorous qualitative methods 27, 28. The value of some studies to this review (CASP question 10) was unclear or low because they presented mixed results from both mothers and healthcare providers and some only had a small section about testing. The relationship between the researcher and participants and ethical issues were poorly considered in general.

**Figure 3:**
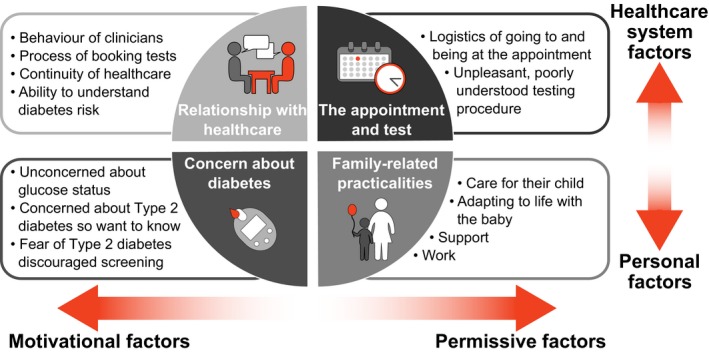
Summary of the themes and subthemes of influences on attendance at postpartum glucose testing after gestational diabetes. GDM, gestational diabetes.

Barriers and facilitators to attending screening after GDM were translated into four themes (relationship with health care, the appointment and test, family‐related practicalities and concern about diabetes) and 13 subthemes (behaviour of clinicians, process of booking tests, continuity of health care, ability to understand diabetes risk, logistics of going to and being at the appointment, testing procedure unpleasant or did not understand its purpose, care for their child, adapting to life with the baby, support, work, unconcerned about discovering their glucose status, concern regarding a diabetes diagnosis and fear of diagnosis of diabetes discouraged screening). The themes and subthemes are described below. Although not discrete categories, we organized the themes into quadrants according to the degree to which they related to the healthcare system or were personal factors, and the degree to which they supported attendance (permissive factors) or influenced attitudes towards testing (motivational factors). This is summarized in Fig. 3 and the studies that contributed to each theme are shown in Table S3. Influences were reported from the perspective of GDM‐affected participants but not all participants were influenced by each factor.

### Relationship with health care

Participants’ interaction with the healthcare system influenced their intentions towards screening.

The behaviour of clinicians could conflict with or reinforce prioritization of screening. Pregnancy and postpartum care could imply that GDM and the associated diabetes risk were not important after delivery, therefore there was no need for further testing. For example, the message that GDM would resolve after delivery could appear inconsistent with messages about postpartum screening: ‘… my diabetes midwife said it normally goes away after the pregnancy so I didn't get anything afterwards’ 29. Women were also confused because glucose monitoring and dietary restrictions stopped immediately: ‘I sat there in the hospital eating a big huge piece of chocolate cake …’ 30. Furthermore, some clinicians had ‘no time’ for glucose testing 31 but focused on the baby or non‐diabetes maternal care at postpartum appointments. By contrast, clinicians ‘promoting’ follow‐up 32 helped women to understand its importance, for example, ‘I think that [postnatal follow‐up] was explained to me both pre and post that that needed to happen. It was explained by both the hospital and the GP’ (screened) 33.

Participants additionally commented on the process of booking tests. Many were surprised to discover that it was their responsibility rather than doctors’ and that missed appointments were not chased. They often needed to act on generic information, such as ‘… [the leaflet] said it was something I was supposed to take care of myself…’ (screened) 31. Although many did arrange the test, some considered that invitations and reminders should come from the doctors: ‘Well, it would be a lot easier if I got a letter that said, now it's time – like they do for that cervix cancer screening’ [screened] 31; proactive clinicians encouraged attendance: ‘… [my doctor] even wrote it down in my insurance booklet’ (screened) 25. Participants would be reassured to know that GPs were involved in this part of their care because ‘… You tend to forget … so much occurs after the childbirth’ 34. At an extreme, some women perceived that their GP did not know about routine follow‐up care after GDM, ‘Even for blood test I had to tell him I have to do a blood test for diabetes’ (screened) 33, or explicitly gave incorrect advice. One participant concluded that ‘[GPs] don't really understand it, GDM, at all’ (screened) 33.

In addition, continuity of health care was frequently discussed. Some women were distressed by lack of continuity: ‘… You see all different [doctors] and then they didn't have my record and … everybody just seems so confused here, like they don't know what's going on with their patient’ (attended visit) 35. Conversely, consistency in relationships meant that they knew and trusted their clinicians, and could feel safe with predictable appointments: ‘It meant a lot to me that I didn't have to see a new person every time I was there. That would definitely have made me feel all confused – it wouldn't have been fun at all …’ 31. Fragmented care was particularly obvious between pregnancy and returning to the GP postpartum, where Bernstein *et al*. referred to a ‘chasm between specialities’ and ‘professional silos’ 36. Consequently, some needed to take on the role of ‘information broker’ 33 and communicate their pregnancy history with their GP; electronic medical records were not sufficient 33, 36. Additionally, Bennett *et al*. reported that relationships built with administrative staff facilitated follow‐up: ‘… when I called to reschedule [the clerk]'s like, “Oh, I was hoping you'd bring the baby so I could see him.” So I told her I'd bring him’ (screened) 35.

Finally, clinicians played an important role in the ability to understand diabetes risk. A lack of patient‐focus prevented participants from asking questions about GDM because there was only time for clinicians’ agenda in consultations, ‘She [GP] basically said don't eat any carbs, any sugar, don't eat any fruit … I was sort of like a bit overwhelmed. I came home and I just cried because there is nothing I can eat now…’ (not screened) 33, or because it was explained using medical terminology that they could not understand 33. Some clinicians were too keen to refer them to websites and/or leaflets. Inability to learn about GDM could leave women anxious and uninformed about their risk of diabetes or the need for screening. Several identified the need for ‘good education antenatally as well as once you've had the baby [and] your brain's working again …’ 32.

### The appointment and test

Practical aspects of both the appointment and the glucose test itself affected opportunity to attend.

Logistics of going to and being at the appointment could create several barriers to attendance. These included the appointment time, needing to travel long distances or needing to use public transport, which one participant experienced all of: ‘It was a long and tiring day and I was exhausted when I got back home’ 37. Some factors were inherent to current OGTT procedures such as the long appointment: ‘because it took 2 h of my time I kept putting it off’ 28. Furthermore, lack of health insurance or the ability to pay for testing prevented attendance: ‘I don't really need [testing] … only because of how much it costs, since we are in a terrible financial position’ (not screened) 26.

Women found the testing procedure unpleasant or did not understand its purpose therefore wanted to avoid having to go through it. In particular, many reported that fasting then drinking a glucose solution made them feel ill, and some disliked needles. Some respondents indicated that they did not understand how the test worked, meaning one participant ate breakfast so had to come back another time 35, and another questioned the procedure saying, ‘… How can you give somebody sugar to drink and then you're going to have to test it? They're definitely going to find the sugar’ 36. Several suggested using more pleasant tests 28.

### Family‐related practicalities

Respondents reported various personal challenges to attending screening tests. As illustrated by the response ‘… everything is about your baby …’ 31, these tended to relate to children. Bernstein *et al*. said that ‘most women opt to plan activities around the needs of the newborn, not around the needs of the medical care system’ 36, therefore if the two were not compatible, they did not attend.

Mothers said that needing to care for their child prevented screening attendance: ‘I don't think there was anything that made me hesitate other than, you know, life with a newborn and two other children …’ 38. Several mentioned their schedules: some reported that a new baby led to a lack of a schedule, ‘… [getting things] done happens in the window of opportunity on the spur of the moment’ 32, whereas others struggled around feeding and sleep routines. Importantly, the clinic was not seen to be a suitable place to wait with children or to breastfeed. Bennet *et al*. reported that few women brought their children to the test 35; when others spoke about the need to find childcare, it appeared that bringing them was not considered an option (due to the anticipated challenges of the waiting room and during the procedure). ‘A “separate room to facilitate breast feeding, toys for kids, nappy changing facilities” at the testing centres may also facilitate screening attendance’ 28. This theme was more important in unusual or unexpected circumstances: ‘I guess [I didn't come be]cause [I was] seeing the baby [at the hospital] every day … It's the only thing I did …’ (not screened) 35.

Unsurprisingly, adapting to life with the baby was difficult and women described feeling ‘just tired … because I'm burnt out, frustrated’ (not screened) 35 and that ‘life is stressful. With a new baby, mum gets no sleep and has no energy and … may be feeling overwhelmed’ 37. In the context of ‘trying to get showers in and get food in is an issue right now’ (screened) 35, mothers’ own health and arranging testing were forgotten or simply too much, although many intended to go at a later date or when things were more under control, ‘I had no time to go … Always I tell I do it tomorrow … But I do not gone again, because I have to do another duty …’ (not screened) 26.

Furthermore, the support that women received at home affected their ability to take time away from childcare and attend testing: several mentioned that their husbands or parents had looked after the children, whereas others did not have this option. One participant explained that ‘Because of my children, I cannot go out much … There is no one to keep an eye on them while I'm gone’ (not screened) 26.

Finally, the need to work presented a further barrier to attendance because women were not able to take time away for the test: ‘I couldn't leave work because they could take it away and I knew the situation I was in, I needed to work’ 36, and it presented another demand on their time: ‘… I've been running around trying to get stuff done before I go back to work’ (screened) 35.

### Concern about diabetes

Lastly, participants’ level of concern regarding diagnosis of diabetes was a key factor affecting motivation to attend screening.

Some participants were unconcerned about discovering their glucose status so were not motivated to attend screening. This represented apathy (‘could not be bothered’ and ‘having a slack attack’ 37) or a lack of urgency 33. Others were untroubled by the possibility of a diabetes diagnosis because they did not deem themselves to be at risk. One denied her diagnosis, which was outlined in her medical record, saying ‘My glucose level was not too high. It wasn't GDM …’ (not screened) 25. Some had evaluated that they did not have diabetes due to reassuring results of self‐monitoring that they continued postpartum, concluding ‘everything is normal’ (not screened) 35, and because they felt healthy or were ‘very careful and compliant’ with lifestyle recommendations (not screened) 25. Other women were unconcerned but were nevertheless tested as screening coincided with other aspects of postpartum care or marked ‘closure with their care’ 35.

Concern regarding a diabetes diagnosis and understanding the need for management most often encouraged screening. In particular, understanding the significance of diabetes was a motivator to attend, ‘… so I am afraid of diabetes … That's why I'm screening’ (screened) 25. This could be reinforced through knowing friends and family with diabetes, or their own experience: one participant considered the implications of a diagnosis very seriously, saying ‘… I would have to ask for counselling or something to help me cope with that …’ 36. Additionally, plans for future pregnancies motivated some to be tested ‘… to avoid any complications that might jeopardize her ability to do this successfully’ 38. Abnormal results of self‐monitoring increased concern about diabetes risk and stimulated formal screening.

Occasionally, women's fear of diagnosis of diabetes discouraged screening as they tried to hide from it: ‘It's, like, oh my gosh, I don't want to have it. And so, I guess, in my mind, it's been, if I don't get checked, maybe I won't develop it’ 38.

### Recommendations for promoting postpartum testing

In light of the findings, we developed recommendations for approaches to encourage attendance at glucose testing, both at 6 weeks postpartum and beyond (Table 2). These reference  BCTs and are directed at both women with GDM (such as 5.1 Information about health consequences) and clinicians or the healthcare system (such as 12.1 Restructuring the physical environment) 23. We had high confidence in three, moderate confidence in six and low confidence in one recommendation(s) in accordance with the GRADE‐CERQual assessment; this is summarized in Table 2 and fully explained in Table S4.

**Table 2:** Ten recommendations for promoting postpartum glucose testing after gestational diabetes, and our confidence in each recommendation made using the GRADE‐CERQual approach

Recommendation	Behaviour change techniques relating to recommendation 23	Confidence in evidence and explanation
Relationship with health care		
Educate clinicians to, and how to, promote screening throughout GDM and subsequent care	1.1 Goal setting (behaviour) 4.1 Instruction on how to perform the behaviour 9.1 Credible source	High: lack of information (during pregnancy and postpartum) and seemingly conflicting advice about postpartum screening from clinicians were clearly reported, whereas the opposite encouraged screening
Implement recall systems for postpartum testing from general practice or obstetric care, and send reminders to non‐responders/for missed appointments	1.4 Action planning 1.6 Discrepancy between current behaviour and goal 2.2 Feedback on behaviour	High: benefits or anticipated benefits of invitations and reminders were reported in many studies
Establish standard protocols for communicating gestational diabetes history within the healthcare system	12.5 Adding objects to the environment [for clinicians only]	Moderate: there was a clear need to ensure sharing of patient history within the healthcare system, which would improve follow‐up care; one benefit may be improved screening uptake
Promote patient‐centred approaches to care in order to facilitate building relationships and opportunities to ask questions	4.1 Instruction on how to perform the behaviour [for clinicians only] 9.1 Credible source	Moderate: improving experience of care would make it more pleasant and may improve screening attendance (directly or indirectly)
The appointment and test		
Make clinics more child and nursing‐friendly, and encourage mothers to bring children to appointments	1.4 Action planning 12.1 Restructuring the physical environment 12.5 Adding objects to the environment	Moderate: it is clear that clinics/long appointments are not considered suitable places to bring children but how to improve this was rarely discussed in the studies
Seek innovative, personalized options to make it easier for hard‐to‐reach women to attend testing (e.g. drop‐ins, alternative locations)	12.1 Restructuring the physical environment	Moderate: too inconvenient appointments discouraged testing but the studies did not clearly suggest alternatives
Utilize more pleasant, less time‐consuming testing procedures and protocols	None	Moderate: OGTTs discourage screening; a shorter test without fasting or a glucose drink is desired and may increase uptake
Personal and family‐related practicalities		
Schedule postpartum glucose testing to coincide with other postpartum check‐ups (both mothers’ and children's appointments)	10.5. Social incentive 10.7. Self‐incentive	Low: glucose tests were difficult to attend; it is assumed that combing them with appointments that women are more motivated to attend would facilitate attendance
Concern about diabetes		
Educate women about the purpose of screening and how the procedure works	4.1 Instruction on how to perform a behaviour 5.1 Information about health consequences	High: often knowledge of the purpose of screening increased attendance; apathy and fear of diagnosis were barriers but could be reduced through education
Educate women that postpartum self‐testing, behaviour compliance or one negative test result is not sufficient to rule out Type 2 diabetes in the long term	5.1 Information about health consequences	Moderate: many studies explored how postpartum self‐testing influenced concern about diabetes; education that this is not sufficient to rule out diabetes could increase screening attendance

GDM, gestational diabetes; OGTT, oral glucose tolerance test.

## Discussion

Through a synthesis of qualitative studies, we have shown how multiple healthcare and personal factors influence attendance at postpartum glucose testing after GDM. These factors could act as both barriers and facilitators (although barriers were dominant in the studies we included), and some influenced practical aspects, whereas others affected desire or motivation to attend. Those with high intention for testing may be able to overcome certain logistical barriers and attend, whereas these same barriers may stop less motivated women. We focused on postpartum testing yet several influences were clearly being established during pregnancy. Accordingly, we have identified and assessed our confidence in multiple approaches to increase attendance.

### Strengths and limitations

We completed a rigorous literature search and qualitative synthesis as a multidisciplinary team for this review. To minimize our bias as researchers, we discussed the analysis and used CASP and CERQual checklists when evaluating the quality of studies that contributed to the synthesis and our confidence in the resulting recommendations. We utilized the BCT taxonomy to describe strategies to promote screening in this population. Additionally, we included perspectives from different populations and healthcare systems and found influences that could be relevant to any setting. For example, the cost of testing mostly related to paying for the test yet in settings with free health care, costs associated with travel (e.g. parking charges) may be a barrier.

Some of the 16 papers we included were poor quality and/or only contributed a small amount to the review findings. There was inevitable selection bias, whereby people with stronger views were more likely to participate than those without. However, participants included both women who had attended screening and those that had not. Our interpretations were also limited by the data that were reported: we sought to focus on attendance at screening rather than postpartum care seeking more generally, but were not always able to distinguish between the two. Similarly, use of OGTT, FPG or HbA_1c_ tests was not reported, although descriptions from participants suggest most were offered an OGTT. Fewer studies specifically discussed how to increase screening attendance, therefore our recommendations were primarily suggestions of how to overcome barriers. In addition, it was difficult to identify patterns in influences. For example, although some will be similar, it is likely that influences will vary between the first test at 6 weeks postpartum and diabetes screening several years after pregnancy, yet it was also often unclear how long after pregnancy participants referred to. We were also not able to consider individual‐level interactions such as whether first‐time mothers were more influenced by certain factors than experienced mothers. Although participants criticized or identified gaps in their care (or praised the system), the extent to which this contributed to their decision to attend screening or not is not clear.

### Comparison with other studies

Although we analysed the data using thematic synthesis rather than a framework‐based approach, the influences we identified operated in a way similar to those described in the COM‐B model of behaviour 41. On the one hand, we identified motivational influences: emotions such as worry about diabetes and relationships with health care. On the other hand, our permissive themes could be described as opportunity and capability to attend, where we consider external factors that prompt or inhibit screening, and psychological and physical potential.

Our findings echo many of those identified by Van Ryswyk *et al*. 15. Although their review covered the wider context of healthcare seeking after GDM, we were able to develop understanding specifically related to postpartum testing in addition to attending appointments. For example, we were able to explain their finding that ‘Some women felt a sense of postpartum abandonment after the intensive antenatal management of their GDM …’ (p. 144) and how it related to postpartum testing. Additionally, factors relating to time were often the most frequently reported barriers to attendance in surveys 28, 38, 42.

Several of the influences that we identified were also recognized by healthcare providers, reported in a literature review assessing clinicians’ views towards postpartum testing 43 and by three of the studies analysed here 32, 36, 37. In particular, clinicians considered that mothers should take more responsibility for their diabetes risk, and they were hindered by incomplete knowledge of their patients’ pregnancy history. Although there is agreement that long‐term follow‐up should take place in primary care, there is inconsistency and lack of clarity regarding responsibility for short‐term follow‐up 43, 44. In line with many clinical guidelines, one survey found that primary care was GPs’ and mothers’ preferred place for postpartum testing 42, yet others reported that if a postpartum test ordered by obstetric care was positive, the patient would have been discharged by the time the result was received and secondary care would be unable to follow‐up 36.

### Implications

An important aspect of many of the recommendations is developing women's understanding of both the necessity and procedure of screening, therefore increasing capability and motivation. Positively, many report awareness of the risk of developing Type 2 diabetes 29, 31, 33, 34, 36, 38 but this did not always impact sufficiently on screening attendance. We, therefore, suggest reinforcing the following key messages to address different perspectives and promote screening, without false assurance or exaggerated concern:

- Having had GDM means you are at a higher risk of developing of Type 2 diabetes, which is a serious condition (addressing apathy).
- We want to diagnose diabetes early (apathy) but, typically, it is initially asymptomatic so formal testing is needed. This differs from the glucose monitoring in pregnancy (self‐testing reassurance).
- We can manage diabetes effectively through medication and changes to lifestyle. Early diagnosis improves long‐term outcomes (fear) and knowing your diagnosis enables proactive management of your health (using proactiveness).
- Blood glucose control usually returns to normal after delivery but this needs to be checked postpartum as part of routine GDM follow‐up (informing risk perception).
- Diabetes can affect subsequent pregnancies (tested for other reasons).

Because sharing this information is already included in many guidelines about diabetes, communication must be optimized to increase understanding. It could be provided as a guide through and beyond GDM using specifically developed wording. It could refer back to experiences from pregnancy to improve relatability and understandability (e.g. postpartum testing could be described in relation to pregnancy OGTTs). This information could be available to pregnant women and their clinicians in order to reduce fragmentation of care and confusion over who is responsible for testing.

Additionally, we suggest several changes to healthcare provision that may increase screening. Aside from improving clinicians’ awareness of agreed protocols, steps could be taken to adapt usual practice to remove some barriers to screening. Systematic reviews have found that reminders and recall systems, such as phone calls or letters to both mothers and GPs, are associated with higher uptake of screening than usual care 10, 11. However, a recent evaluation from the Australian National Gestational Diabetes Register, a much larger cohort, suggested that mail‐outs had negligible impact on postpartum and annual follow‐up 45. Although the reasons for this warrant investigation, the authors suggest that more personalized, local invitations might be more effective than national recall. Furthermore, one study reported mothers’ preference for electronic reminders, particularly text messages (sent by the study team) 46. Clinicians also had positive views towards reminders 43 and some advise their patients to have a blood test in the month of their child's birthday (R. Fox, personal communication). It should be considered whether combining glucose testing with other appointments, such as newborn check‐ups, child vaccination schedules or cervical cancer screening in the long‐term, could be both manageable for general practice and offer benefits to women.

Our qualitative synthesis also supports the need for further consideration of more acceptable screening tests due to the length and inconvenience of the OGTT and the need to fast then sugar load. The HbA_1c_ test is an accurate measure of chronic glycaemia in the general population that requires one non‐fasting blood sample 47 although it is not suitable for use shortly after pregnancy and questions about its sensitivity remain 48, 49. Similar to the change in the NICE guidelines in 2015 8, recent guidelines in Australia and New Zealand have recommended HbA_1c_ testing after the postpartum period. Small‐scale analyses suggest that HbA_1c_ testing can have a higher uptake than OGTTs, yet uptake remains suboptimal in the long‐term 50, 51. Our findings provide additional evidence that this could reduce some motivational barriers to screening and make it easier to complete alongside other tests or appointments. In addition, novel strategies such as very early postpartum testing (e.g. before leaving hospital) could be considered. Although less accurate than a test at 6 weeks, very high uptake can be achieved and therefore high‐risk women can be identified for targeted follow‐up 52. Further research over longer periods is needed to evaluate the benefits and harms of increased use of other tests.

### Conclusion

After a pregnancy with GDM, difficulties associated with attending appointments and a focus on the family can affect women's ability to attend glucose testing postpartum and in the long‐term. Concern about risk of developing diabetes and experiences of health care can increase or limit intentions towards testing. Alongside clearer education about GDM, we have suggested that amendments to healthcare provision during and after pregnancy will decrease barriers to testing. Higher uptake will enable earlier management of diabetes and improve long‐term outcomes.

## Funding sources

R.D. is funded by a PhD studentship from the National Institute for Health Research (NIHR) School for Primary Care Research (SPCR; SPCR‐S‐S102). This paper presents independent research funded by the NIHR SPCR. The views expressed are those of the author(s) and not necessarily those of the NIHR, the NHS or the Department of Health. R.W. is funded by an NIHR Academic Clinical Fellowship. S.G. is supported by the Medical Research Council (MC_UU_12015/4). S.G. is an NIHR Senior Investigator. The University of Cambridge has received salary support in respect of S.G. from the NHS in the East of England through the Clinical Academic Reserve. J.U‐S. is funded by a Cancer Research UK Cancer Prevention Fellowship (C55650/A21464).

## Competing Interests

None.

## Supporting information


**Table S1**. Medline search strategy.
**Table S2**. Findings from the Critical Skills Appraisal Programme (CASP) checklist.
**Table S3**. Studies contributing to each theme.
**Table S4**. CERQual qualitative evidence profile of recommendations for promoting attendance at diabetes screening after gestational diabetes.
