# Supplementary material for: Women's views on screening for Type 2 diabetes after gestational diabetes: a systematic review, qualitative synthesis and recommendations for increasing uptake
Source: Diabet Med. 2019 Jul 22;37(1):29–43. doi: 10.1111/dme.14081 (PMC6916174; doi:10.1111/dme.14081)
Supplement: Supplementary file 1 — Table S1. Medline search strategy. Table S2. Findings from the Critical Skills Appraisal Programme (CASP) checklist. Table S3. Studies contributing to each theme. Table S4. CERQual qualitative evidence profile of recommendations for promoting attendance at diabetes screening after gestational diabetes. [file DME-37-29-s001.pdf]

Supplementary material

TABLE S1: Medline search strategy

1.	type 2 diabetes.mp. or Diabetes Mellitus, Type 2/
2.	T2DM.mp.
3.	NIDDM.mp. or Diabetes Mellitus, Type 2/
4.	non insulin dependent diabetes.mp.
5.	glucose tolerance.mp.
6.	insulin resistance.mp. or Insulin Resistance/
7.	1 or 2 or 3 or 4 or 5 or 6
8.	gestational diabet*.mp.
9.	diabetes in pregnancy.mp.
10.	Pregnancy/ or pregnancy.mp.
11.	type 2 diabet*.mp.
12.	10 and 11
13.	gestation*.mp.
14.	11 and 13
15.	postpartum diabet*.mp.
16.	postpartum.mp. or Postpartum Period/
17.	8 or 9 or 12 or 14 or 15 or 16
18.	prevent*.mp.
19.	progress*.mp.
20.	develop*.mp.
21.	advanc*.mp.
22.	incidence.mp. or Incidence/
23.	avoidance.mp.
24.	prohibit.mp.
25.	establish.mp.
26.	health promotion.mp. or Health Promotion/
27.	Exercise/ or exercise.mp.
28.	active living.mp.
29.	metformin.mp. or Metformin/
30.	weight.mp. or "Weights and Measures"/
31.	risk factors.mp. or Risk Factors/
32.	Insulin/ or insulin.mp.
33.	exercise therapy.mp. or Exercise Therapy/
34.	intervention.mp.
35.	interven*.mp.
36.	yoga.mp. or Yoga/
37.	postnatal.mp.
38.	diet.mp. or Diet/
39.	healthy eating.mp. or Healthy Diet/
40.	behaviour.mp.
41.	physical activity.mp. or Exercise/
42.	lifestyle.mp. or Life Style/
43.	manag*.mp.
44.	screening.mp. or Mass Screening/
45.	hypoglycaemic agents.mp.
46.	hypoglycaemics.mp.
47.	health promotion.mp. or Health Promotion/
48.	medication.mp.
49.	medical therapy.mp.
50.	rate.mp.
51.	predictor*.mp.

52. risk*.mp.
53. factor*.mp.
54. 18 or 19 or 20 or 21 or 22 or 23 or 24 or 25 or 26 or 27 or 28 or 29 or 30 or 31 or 32 or 33 or 34 or 35 or 36 or 37 or 38 or 39 or 40 or 41 or 42 or 43 or 44 or 45 or 46 or 47 or 48 or 49 or 50 or 51 or 52 or 53
55. follow-up.mp.
56. postpartum.mp. or Postpartum Period/
57. qualitative.mp.
58. Interview/ or interview.mp.
59. focus group*.mp.
60. health service.mp. or Health Services/
61. belief*.mp.
62. opinion*.mp.
63. survey.mp.
64. 54 or 55 or 56 or 57 or 58 or 59 or 60 or 61 or 62 or 63
65. 7 and 17 and 64

TABLE S2: Findings from the Critical Skills Appraisal Programme (CASP) checklist

Study		1. Clear statement of aims?	2. Qualitative methodology?	3. Appropriate research design?	4. Appropriate recruitment strategy?	5. Suitable data collection?	6. Researcher-participant relationship considered?	7. Ethical issues considered?	8. Rigorous data analysis?	9. Clear findings?	10. Valuable to us?	Score (/10)
**Soares 2006**		🟡	🟢	🟡	🟡	🟡	🔴	🟡	🔴	🔴	🔴	3.5
**Bennet 2011**		🟢	🟢	🟢	🟢	🟢	🔴	🟡	🟢	🟢	🟢	8.5
**Sterne 2011**		🟢	🟢	🟡	🟢	🔴	🔴	🟡	🔴	🟢	🟡	5.5
**Lie 2013**		🟢	🟢	🟢	🟢	🟢	🔴	🟡	🟢	🟢	🟡	8.0
**Abraham 2014**		🟢	🟢	🟢	🟡	🟢	🔴	🟡	🟢	🟢	🔴	7.0
**Morrison 2014**		🟢	🟡	🟡	🟢	🟡	🔴	🟢	🟢	🟡	🟡	6.5
**Paez 2014**		🟢	🟢	🟢	🟢	🟢	🔴	🟡	🟢	🟡	🟢	8.0
**Kilgour 2015**		🟢	🟢	🟢	🟢	🟢	🟡	🟢	🟡	🟢	🟢	9.0
**Nielsen 2015**		🟢	🟢	🟢	🟢	🟢	🟢	🟢	🟢	🟢	🟢	10.0
**Bernstein 2016**		🟢	🟢	🟢	🟡	🟡	🔴	🟡	🟡	🟢	🟡	6.5
**Campbell 2017**		🟢	🟢	🟢	🟡	🟢	🟢	🟢	🟢	🟢	🟡	9.0
**Pennington 2017**		🟢	🟢	🟢	🟢	🟢	🔴	🟡	🟢	🟢	🟡	8.0
**Rafii 2017a**		🟢	🟢	🟢	🟢	🟢	🔴	🟡	🟡	🟢	🟡	7.5
**Rafii 2017b**		🟢	🟢	🟢	🟢	🟢	🟡	🟢	🟢	🟢	🟢	9.5
**Svensson 2017**		🟢	🟢	🟢	🟡	🟢	🔴	🟢	🟢	🟢	🔴	7.5
**Zulfiqar 2017**		🟢	🟢	🟢	🟢	🟢	🟡	🟡	🟡	🟢	🔴	7.5
Score frequency	Yes	15	15	13	11	12	2	6	10	13	5	
	Unclear	1	1	3	5	3	3	10	4	2	7	
	No	0	0	0	0	1	11	0	2	1	4	

Green dot: yes (1 point); yellow dot: can't tell/unclear (0.5 points); red dot: no (0 points)

<u>TABLE S3</u>: Studies contributing to each theme

		Relationship with healthcare				Appointment and test		Family-related practicalities				Concern about diabetes		
**Study**	**CASP score**	Behaviour of clinicians	Process of booking tests	Continuity of healthcare	Ability to understand diabetes risk	Unpleasant, poorly understood testing procedure	Logistics of the appointment	Care for their child	Adapting to life with the baby	Work	Support	Unconcerned about glucose status	Concerned about T2D so want to know	Fear of T2D discouraged screening
Soares 2006	3.5		∘				∘		∘					
Bennet 2011	8.5			⬤			◯	⬤	⬤	⬤	⬤	⬤	⬤	⬤
Sterne 2011	5.5	•	•		•	•	•	•	•		•	∘	•	∘
Lie 2013	8.0	●	●									○	○	○
Abraham 2014	7.0	•		∘	•							∘	∘	
Morrison 2014	6.5	•												
Paez 2014	8.0	●	●	○		○	●	○	●	○	●	●	●	●
Kilgour 2015	9.0	⬤	⬤	⬤	⬤		◯	◯	◯			◯	◯	
Nielsen 2015	10.0	⬤	⬤	⬤	⬤				⬤			⬤	⬤	
Bernstein 2016	6.5			•	∘	•	•		•	•	•	∘	•	
Campbell 2017	9.0			⬤	⬤		⬤	◯	⬤		◯	⬤	◯	
Pennington 2017	8.0		●	●	○	●	○		●			○		
Rafii 2017a	7.5		○		●	○	●	●			○	●	○	●
Rafii 2017b	9.5		⬤		⬤				◯			⬤	⬤	◯
Svensson 2017	7.5	○	●	○	●									
Zulfiqar 2017	7.5	○	○									●	●	

Large dot: CASP score ≥8.5, medium dot: 7.5–8.0 (median=7.75), small dot: ≤7.0.

Open dots indicate where a study briefly contributes to the theme, or lists the theme

TABLE S4: CERQual qualitative evidence profile of recommendations for promoting attendance at diabetes screening after gestational diabetes

Objective: To systematically synthesise the literature focussing on the views of women with a history of GDM on attendance at postpartum glucose testing
Perspective: Views, experiences and ideas of any women who have had GDM during any previous pregnancy
Included studies: Studies that examine women's postpartum experiences following GDM relating to attendance at postpartum glucose testing

	Review recommendation	Studies directly contributing to the recommendation	Assessment of methodological limitations	Assessment of relevance	Assessment of coherence	Assessment of adequacy	Overall CERQual assessment of confidence	Explanation of CERQual assessment
**Relationship with healthcare**								
**1**	Educate clinicians to, and how to, promote screening throughout GDM and subsequent care	Abraham, Campbell, Kilgour, Lie, Morrison, Nielsen, Paez, Rafii a, Sterne, Svensson, Zulfiqar	Minor concerns: the highest quality studies contributed most to informing this recommendation	Minor concerns: these findings addressed attitudes towards screening (rather than general healthcare seeking, which was also sometimes considered)	Minor concerns: for many participants, clinicians played the key part in forming views toward screening	Minor concerns: several studies discussed in detail how women interpreted (lack of) information and others more briefly mentioned this idea	**High confidence**	Lack of information (during pregnancy and postpartum) and seemingly conflicting advice about postpartum screening from clinicians were clearly reported, while the opposite encouraged screening
**2**	Implement recall systems for postpartum testing from general practice or obstetric care, and send reminders to non-responders/for missed appointments	Kilgour, Lie, Nielsen, Paez, Pennington, Rafii a, Rafii b, Sterne, Zulfiqar	Minor concerns: the highest quality studies contributed most to informing this recommendation	No or very minor concerns: these findings clearly addressed attitudes towards arranging the screening test (rather than general healthcare seeking, which was also sometimes considered)	Minor concerns: invitations from clinicians were reported positively; participants wanted reminders; many took control of arranging tests but reported this negatively	Minor concerns: several studies discussed arranging tests: the majority discussed difficulties when they didn't receive support but some discussed invitations and reminders helping	**High confidence**	Benefits or anticipated benefits of invitations and reminders were reported in many studies
**3**	Establish standard protocols for communicating gestational diabetes history within the healthcare system	Bennett, Bernstein, Campbell, Kilgour, Nielsen, Svensson	Minor concerns: four high and two good quality studies contributed to this recommendation; two studies considered the researcher-participant relationship so this may have influenced the discussion about the healthcare system in the others	Minor concerns: these findings were relevant to postpartum follow-up including screening	Moderate concerns: six studies clearly discussed fragmented care and women as information brokers, which lead to postpartum abandonment and getting lost between specialities; one explained how this discouraged screening attendance	Moderate concerns: data regarding women's discussion of continuity of care were rich but explanations on the consequences for screening were sparse	**Moderate confidence**	There was a clear need to ensure sharing of patient history within the healthcare system, which would improve follow-up care; one benefit may be improved screening uptake
**4**	Promote patient-centred approaches to care in order to facilitate building relationships and opportunities to ask questions	Links to healthcare provision in general; specifically Abraham, Bennett, Campbell, Kilgour, Nielsen	No or very minor concerns: the studies that directly contributed to this recommendation were the highest quality	Minor concerns: these findings were relevant to postpartum follow-up including screening	Moderate concerns: it is clear and logical that patient-centred care improves healthcare experience but less clear from these studies that screening attendance would increase as a result	Moderate concerns: few studies contributed directly to this recommendation, however, all of the studies that discuss the healthcare system inform patient-centred care in some way	**Moderate confidence**	Improving experience of care would make it more pleasant and may improve screening attendance (directly or indirectly)
**The appointment and test**								
**5**	Make clinics more child and nursing-friendly, and encourage mothers to bring children to appointments	Bennett, Kilgour, Paez, Rafii a, Sterne	Moderate concerns: four studies were very high quality but Sterne contributed most to this theme and had many methodological limitations	Minor concerns: these findings were relevant to postpartum follow-up and screening appointments	Moderate concerns: it was clear that many women did not consider taking the baby to the appointment so struggled to go if they couldn't find childcare; some participants suggested improving clinic environments	Moderate concerns: data about the need for childcare were rich, but there were fewer data about changing clinic environments and bringing children	**Moderate confidence**	It is clear that clinics/long appointments are not considered suitable places to bring children but how to improve this was rarely discussed in the studies
**6**	Seek innovative, personalised options to make it easier for hard-to-reach women to attend testing (eg. drop-ins, alternative locations)	Bennett, Bernstein, Campbell, Paez, Rafii a, Rafii b, Sterne	Minor concerns: several high quality studies contributed most to informing this recommendation	Minor concerns: these findings were relevant to postpartum follow-up and screening appointments	Moderate concerns: how easy/convenient it was to attend the test affected uptake, highlighting this as an area for improvement; one study suggesting home testing	Moderate concerns: data about the inconvenience of testing were rich but how to improve it was rarely reported	**Moderate confidence**	Too inconvenient appointments discouraged testing but the studies did not clearly suggest alternatives

**7**	Utilise more pleasant, less time-consuming testing procedures and protocols	Bernstein, Paez, Pennington, Rafii a, Sterne	Moderate/minor concerns: two of the five studies contributing to this theme were low quality but this is not expected to have a large impact on this recommendation	No or very minor concerns: these findings clearly addressed attitudes towards arranging the screening test	Moderate concerns: the need to fast, drink a glucose drink and wait were clear barriers to the OGTT and alternative tests were suggested, but no studies showed increased attendance using alternative tests	Minor concerns: the data provide a clear understanding of how OGTTs discourage attendance	**Moderate confidence**	OGTTs discourage screening; a shorter test without fasting or a glucose drink is desired and may increase uptake
**Family-related practicalities**								
**8**	Schedule postpartum glucose testing to coincide with other postpartum check-ups (both mothers' and children's appointments)	Links to inconvenience of appointments and motivation in general; specifically Bennett, Nielsen, Rafii a, Rafii b	No or very minor concerns: the studies that directly contributed to this recommendation were the highest quality	No or very minor concerns: these findings clearly addressed attitudes towards screening and arranging the test	Moderate concerns: participants attended appointments for other reasons (eg. for vaccinations or to discuss contraception) and Rafii b describes 'accidental screening', therefore we only assume that combined appointments are more convenient and worth attending	Major concerns: only a few studies contributed to this theme, plus general inconvenience of appointments and motivation to attend	**Low confidence**	Glucose tests were difficult to attend; it is assumed that combing them with appointments that women are more motivated to attend would facilitate attendance
**Concern about diabetes**								
**9**	Educate women about the purpose of screening and how the procedure works	Abraham, Bennett, Bernstein, Campbell, Kilgour, Lie, Nielsen, Paez, Rafii a, Rafii b, Sterne, Zulfiqar	Minor concerns: mostly high quality studies contributed to this recommendation	Minor concerns: these findings showed that apathy and fear of diagnosis acted as a barrier to screening and understanding the need for screening as a facilitator to screening attendance specifically	Minor concerns: findings show that knowledge about the purpose of screening increased attendance and so it is clear and logical that education of women on the purpose of screening should increase attendance	Minor concerns: several studies discuss the themes contributing to this recommendation in detail	**High confidence**	Often knowledge of the purpose of screening increased attendance; apathy and fear of diagnosis were barriers but could be reduced through education
**10**	Educate women that postpartum self-testing, behaviour compliance or one negative test result is not sufficient to rule out T2D in the long term	Bennett, Bernstein, Kilgour, Lie, Nielsen, Paez, Rafii a, Rafii b	Minor concerns: mostly high quality studies contributed to this recommendation	Minor concerns: these findings were relevant predominantly to postpartum screening, but did include other aspects of post-partum behaviour such as diet	Minor concerns: use of glucometer postpartum consistently discouraged screening attendance in these studies	Moderate concerns: four of the studies discuss the impact of self-testing on screening attendance whilst remaining have sparse findings addressing role of reassurance of postpartum readings and test results generally	**Moderate confidence**	Many studies explored how postpartum self-testing influenced concern about diabetes; education that this is not sufficient to rule out diabetes could increase screening attendance

Recommendations frequently result from findings within multiple themes but have been presented under the primary contributing theme. Only studies directly contributing to the recommendation have been cited

GDM: gestational diabetes; OGTT: oral glucose tolerance test; T2D: type 2 diabetes
